# [18F]Fluoride Positron-Emission Tomography (PET) and [18F]FDG PET for Assessment of Osteomyelitis of the Jaw in Comparison to Computed Tomography (CT) and Magnetic Resonance Imaging (MRI): A Prospective PET/CT and PET/MRI Pilot Study

**DOI:** 10.3390/jcm11143998

**Published:** 2022-07-10

**Authors:** Christian Philipp Reinert, Christina Pfannenberg, Helmut Dittmann, Brigitte Gückel, Christian la Fougère, Konstantin Nikolaou, Sebastian Hoefert

**Affiliations:** 1 Department of Radiology, Diagnostic and Interventional Radiology, University Hospital Tübingen, Hoppe-Seyler-Str. 3, 72076 Tübingen, Germany; christina.pfannenberg@med.uni-tuebingen.de (C.P.); brigitte.gueckel@med.uni-tuebingen.de (B.G.); konstantin.nikolaou@med.uni-tuebingen.de (K.N.); 2 Department of Radiology, Nuclear Medicine and Clinical Molecular Imaging, University Hospital Tübingen, Hoppe-Seyler-Str. 3, 72076 Tübingen, Germany; helmut.dittmann@med.uni-tuebingen.de (H.D.); christian.lafougere@med.uni-tuebingen.de (C.l.F.); 3 Cluster of Excellence iFIT (EXC 2180) “Image Guided and Functionally Instructed Tumor Therapies”, University of Tübingen, 72076 Tuebingen, Germany; 4 German Cancer Consortium (DKTK), Partner Site Tuebingen, 72076 Tuebingen, Germany; 5 Department of Oral and Maxillofacial Surgery, University Hospital of Tübingen, Osianderstr. 2-8, 72076 Tuebingen, Germany; sebastian.hoefert@med.uni-tuebingen.de

**Keywords:** osteomyelitis of the jaw, bone turnover, computed tomography, magnetic resonance imaging, [18F]fluoride, [18F]FDG, positron-emission-tomography, molecular imaging

## Abstract

To investigate imaging features of osteomyelitis of the jaw (OMJ) using [18F]fluoride positron emission tomography (PET) and [18F]fluorodeoxyglucose (FDG)-PET compared with computed tomography (CT) and magnetic resonance imaging (MRI) to assess extent and disease activity. Six female patients (55.3 ± 10.0 years) were enrolled for assessment of symptomatic OMJ. 4/6 patients underwent [18F]FDG-PET/MRI and [18F]fluoride-PET/CT, one patient MRI and [18F]fluoride-PET/CT and another patient only [18F]FDG-PET/MRI. Image analysis was performed by two radiologists, an oral and maxillofacial surgeon, and a nuclear medicine specialist. The extent of affected jawbone was analyzed both qualitatively and quantitatively, including the PET tracer uptake, CT-Hounsfield-Units (HU) and MRI parameters in affected and healthy jawbone. All patients had trabecular sclerosis in the affected jawbone compared to healthy jawbone (560 ± 328 HU vs. 282 ± 211 HU; *p* > 0.05), while 3/6 patients had cortical erosions. Bone marrow edema and gadolinium enhancement were documented in 5/6 patients. In affected jawbone, [18F]fluoride-uptake was increased in all patients compared to healthy jawbone (SUVmean 15.4 ± 4.2 vs. 2.1 ± 0.6; *p* < 0.05), and [18F]FDG-uptake was moderately higher (SUVmean 1.9 ± 0.7 vs. 0.7 ± 0.2; *p* > 0.05). The extent of regions with increased metabolic activity was less than the extent of morphologic changes in all patients. Information on jawbone metabolism and inflammation is different from morphologic changes and therefore has the potential to provide a more accurate and objective assessment of the extent and activity of OMJ.

## 1. Introduction

Osteomyelitis of the jaw (OMJ) is an inflammatory disease of the bone that affects the medullary cavity and extents to the cortical bone, overlying periosteum, and soft tissue [1]. Due to frequent odontogenic infections, the jaws are prone to osteomyelitis, especially in patients with predispositions such as osteoporosis, osteopetrosis, therapeutic radiation, specific drugs such as bisphosphonates, and malignancies [2].

OMJ is divided into acute, secondary chronic, and primary chronic OMJ [1]. Acute OMJ is caused by a bacterial focus due to odontogenic disease, pulpal and periodontal infection, extraction wounds, foreign bodies, or infected fractures and is clinically manifested by suppuration, fistula formation and sequestration. Secondary chronic OMJ may have a suppurative course with abscess or fistula formation and sequestration. Depending on the intensity of the infection and the host bone response, clinical presentation and course can vary significantly. Primary chronic OMJ is a rare nonsuppurative chronic inflammation of unknown origin associated with hyperactivity, hypoactivity, and impairment of immune response. Diagnosis is difficult since the clinical complaints are often nonspecific and swelling, fistulae and one-sided clinical findings are often absent.

Conventional radiological diagnostics are often inadequate to accurately assess the extent of OMJ in symptomatic patients [1,3,4]. Considering functional and aesthetic consequences, early and comprehensive diagnosis of OMJ is imperative to determine appropriate treatment or avoid overtreatment. In this context, conservative nonsurgical therapy is also recommended for primary chronic OMJ [1]. Despite the improvement in the therapeutic management of this disease in the last two decades, diagnosis remains challenging and essential. Clinical parameters such as local pain, swelling, or hypoesthesia of the inferior dental nerve are not often apparent and may vary from patient to patient [5]. Also, no correlation between inflammatory laboratory parameters, such as C-reactive protein, and the course of OMJ has been documented [6,7]. The orthopanoramic view is usually used as an initial examination to assess dental status, but its assessment of bony details, especially trabecular bone, is limited [2]. High-resolution CT with multiplanar slices and 3D reconstructions provides more detail by showing the degree of cortical destruction, osteolysis, fractures, periosteal reactions, and the extent of sclerosis [8]. Molecular imaging may prove useful in diagnosing early subclinical disease by detecting metabolic changes in bone and soft tissue before structural or anatomic changes occur [9]. Several studies using different gamma-emitting radiotracers such as radiolabeled antibodies or gallium-67 (^67^Ga) have contributed to the evaluation of musculoskeletal infections [10,11]. Bone scintigraphy with technetium-99 m labeled bisphosphonates using single photon emission computed tomography (SPECT)/CT has been reported to have high sensitivity for foci of increased bone turnover, however, the underlying increased osteoblast activity is not specific for inflammation [7].

Compared with bone scintigraphy, [18F]FDG-PET has been proposed as a superior method for the diagnosis of osteomyelitis due to its improved spatial resolution and higher specificity, allowing more accurate localization of focal inflammatory lesions characterized by increased glucose uptake [12,13]. In infected bone, [18F]FDG is more readily taken up via glucose transporters, which is related to migrating inflammatory cells, microorganisms, and granulation tissue [14]. [18F]fluoride PET, which has been shown to be useful for the evaluation of medication-induced osteonecrosis of the jaw (MRONJ), can reflect bone metabolism, especially in terms of osteoblastic activity [15]. Compared to conventional bone scintigraphy or SPECT imaging [18F]fluoride PET/CT offers higher sensitivity and specificity with shorter examination times, as well as favorable tracer-kinetic properties with higher bone-soft tissue contrast [16,17,18,19]. It has been reported that [18F]fluoride PET can achieve higher sensitivity than planar 99 mTc-bisphosphonate bone scan in patients with osteoblastic metastases [20].

MRI provides excellent anatomical information of the bone marrow and soft tissues compared with CT, making it suitable for the detection of acute OMJ at an early stage [21]. However, MRI has limited ability to distinguish between edema and infection, and the presence of metal implants may affect diagnostic performance [22]. Although it does not show specific features that allow a definite diagnosis, MRI has a high sensitivity in detecting bone marrow changes, allowing the extent of the disease to be assessed at an early stage [23].

The hybrid imaging modalities PET/CT and PET/MRI allow all of the above information to be obtained in a “one-stop-shop” examination, potentially providing potentially an ideal diagnostic workup for accurate assessment of OMJ.

The aim of this study was to investigate the imaging features of OMJ using [18F]fluoride-PET and [18F]FDG-PET compared with CT and MRI to assess the extent and disease activity.

## 2. Materials and Methods

### 2.1. Patient Cohort

This prospective study was reviewed and approved by the local ethics committee (Project number: 536/2014BO1). All included patients gave their informed consent to the study and to the use of their data for research purposes.

The cohort consisted of six female patients (mean age 55.3 ± 10.0 years) with symptomatic OMJ, including secondary (infectious) and primary (non-infectious) chronic OMJ, who were prospectively enrolled at our institution between 2/2018 and 10/2020. As recommended, OMJ diagnosis was based on the clinical course and previous therapies [1,24,25]. All patient had nonspecific clinical complaints that required accurate diagnosis to localize the OMJ manifestation.

### 2.2. Study Protocol

4/6 patients underwent both [18F]FDG-PET/MRI and [18F]fluoride-PET/CT on the same day, the later scheduled five hours after the first examination. Due to reduced general condition, one patient received only MRI and [18F]fluoride-PET/CT, and another patient received only [18F]FDG-PET/MRI. The time interval between intravenous tracer injections was six hours, otherwise the second measurement with [18F]fluoride would have been too much affected by the first tracer injection. Considering the physical half-life of [18F]FDG of 110 min [26], the calculated concentration of [18F]FDG at the time of [18F]fluoride-PET/CT was reduced to 10%.

### 2.3. [18F]FDG-PET/MRI

Patients fasted for at least six hours before intravenous injection of [18F]FDG. The injected dose was adjusted to patient body weight (1 MBq/kg bodyweight; average dose: 68.4 ± 17.5 MBq). The PET/MRI examinations were performed on a Biograph mMR^®^ scanner (Siemens Healthcare GmbH, Erlangen, Germany). PET acquisition was initiated 40 min after tracer injection from the skull base to the clavicle over one bed position. The PET acquisition time was 30 min. For the generation of a segmentation-based PET attenuation correction map, a 3D T1-weighted spoiled gradient-echo sequence with Dixon-based fat-water separation was acquired. PET was reconstructed using a 3D ordered-subset expectation-maximization algorithm with two iterations, 21 subsets, matrix size 256 × 256, and Gaussian filtering of 4 mm. The following MRI measurements were performed: T2-weighted transversal and coronal turbo spin echo (TSE) sequence, T2-weighted and T1-weighted fast spin echo isotropic 3D sequences allowing multiplanar reformats (sampling perfection with application-optimized contrasts using different flip angle evolution [SPACE^®^], Siemens Healthcare GmbH, Erlangen, Germany), diffusion weighted imaging (DWI), and T1-weighted volumetric interpolated breath-hold examination (VIBE) sequence after intravenous injection of 0.1 mmol/kg gadolinium-based MRI contrast media (GADOVIST^®^, Bayer Vital GmbH, Leverkusen, Germany). The average injected dose of MRI contrast media was 6.0 ± 1.2 mL.

### 2.4. [18F]Fluoride PET/CT

[18F]fluoride PET/CT examinations were performed on a state-of-the art clinical scanner (Biograph mCT^®^, Siemens Healthineers, Erlangen, Germany). PET/CT imaging started 60 min after intravenous application of weight-adapted [18F]fluoride (4 MBq/kg bodyweight; average dose: 284 ± 137 MBq). The patients were positioned in a vacuum mattress to reduce beam-hardening artifacts and motion artifacts. CT examinations were performed without CT contrast agent, using a bone image reconstruction kernel and a slice thickness of 0.6 mm for image reconstruction. PET was acquired from the skull base to the clavicle over one bed position and reconstructed using a 3D ordered subset expectation maximization algorithm (two iterations, 21 subsets, Gaussian filter 2.0 mm, matrix size 400 × 400, and slice thickness 2.0 mm). The PET acquisition time was 15 min.

### 2.5. Qualitative Image Analysis

Image analysis was performed quantitatively and qualitatively by two radiologists, an oral and maxillofacial surgeon, and a nuclear medicine specialist in consensus. The extent of affected jawbone was analyzed qualitatively by CT, MRI and PET, as described below, including morphologic and metabolic features of the jawbone structure and adjacent soft tissue.

The amount of [18F]FDG- and [18F]fluoride tracer uptake was evaluated for each region of the jawbone. Regions showing substantially increased radiotracer uptake above the background radiotracer activity of healthy bone structures were documented. On CT, bone structures were evaluated for periosteal thickening, focal erosions, or medullary sclerosis. On MRI, bone structures and adjacent soft tissues were evaluated by signal alterations on T1-/T2-weighted images as a correlate of bone marrow edema and by increased contrast enhancement on post-contrast T1-weighted VIBE images.

### 2.6. Quantitative Image Analysis

Quantitative analysis included measurements of the PET tracer uptake, CT-Hounsfield Units (HU) and MRI parameters in affected jawbone and healthy jawbone. Two-dimensional regions of interest (ROIs) were manually placed in each region of the jawbone to quantify CT-HU with standard deviation (SD). ROIs were placed in trabecular bone with an average size of 12.7 ± 4.1 mm^2^, carefully excluding osteolytic areas, cortical bone, and teeth. The mean HU of the affected jawbone was calculated by summing all affected regions per patient. Accordingly, the mean HU of healthy jawbone was calculated by the sum of all jawbone regions not affected by OMJ. Semiquantitative indices (SQIs) were calculated for each patient by dividing the mean HU of the affected jawbone by the mean HU of the healthy jawbone.

The ROIs were then copied to MRI, including pre-contrast T1-/T2-weighted and post-contrast T1-weighted sequences. To ensure that only trabecular bone was measured, ROIs were adjusted as necessary.

For PET, radiotracer uptake was quantified by measuring the SUVmean using 50% isocontour volumes of interest (VOIs). As with CT and MRI, VOIs were defined in each region of the affected or healthy jawbone with the same diameter as ROIs. The mean SUV of the affected and healthy jawbone and the SQI were calculated as described above. For [18F]FDG-PET, we additionally calculated the SQI by dividing the SUV_mean_ of the perimandibular soft tissue and the SUV_mean_ of the nasal mucosa as the healthy reference tissue.

### 2.7. Statistics

Statistical analysis was performed using SPSS^®^ Version 22 (IBM Corporation, Armonk, NY, USA). All parameters for the normality were tested by Kolmogorov-Smirnov test. A Mann-Whitney-U test was used to test the differences in PET parameters and CT and MRI features between affected jawbone and healthy jawbone. *p*-values were considered significant at a level of 0.05.

## 3. Results

### 3.1. Clinical Findings

In 5/6 patients, the mandible was affected by OM, while one patient had an OM manifestation in the maxilla. In none of the patients were both jaws affected at the same time. All patients had diffuse recurrent pain, sometimes (but not regularly), swelling, and low mouth opening, sometimes accompanied by regional lymphadenopathy and decreased inferior alveolar sensation. A total of 3/6 patients diagnosed with secondary chronic OMJ had a history of acute suppurative OMJ that was symptomatic despite antibiotic treatment. Primary chronic OMJ was diagnosed in 2/6 patients who achieved therapeutic improvement with nonsteroidal anti-inflammatory drugs (naproxen). In one patient with OMJ, it could not be further specified whether it was primary or secondary OMJ. One patient was treated with ibandronic acid for seven years, but the clinical symptoms were not characteristic for MRONJ; even stage zero (according to Ruggiero [27]) could not be definitely declined. There was no case of chronic recurrent multifocal osteomyelitis (CRMO) or synovitis acne pustulosis hyperostosis osteitis (SAPHO) syndrome. Long-term follow-up of this patient cohort revealed that 4/6 patients had improvement in clinical symptoms, whereas 2/6 patients had stable to worsening symptoms.

### 3.2. Qualitative Analysis

**Figure 1:**
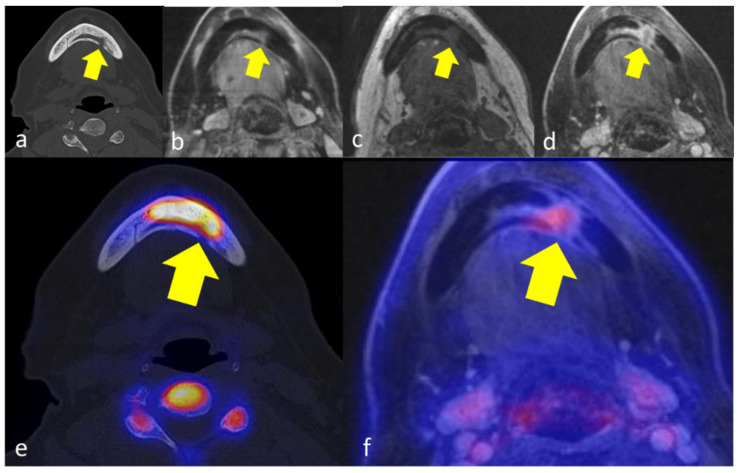
66 years old female patient with secondary chronic OMJ in region 33–35 of the mandible (yellow arrows), showing a diffuse trabecular sclerosis in CT with focal cortical erosion (**a**), corresponding edema in T2-weighted images (**b**), and T1-weighted images (**c**) and increased gadolinium enhancement (**d**). [18F]fluoride-PET/CT reveals markedly increased tracer uptake in the affected jawbone below the extent of sclerosis (**e**) and moderately increased tracer uptake in [18F]FDG-PET/MRI (**f**).

**Figure 2:**
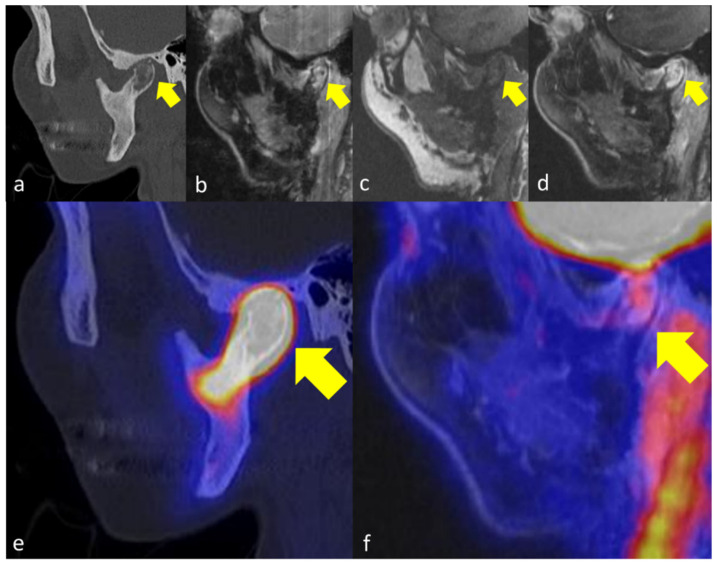
49 years old female patient with primary chronic OMJ in the right mandibular condyle (yellow arrows). CT (**a**) reveals diffuse trabecular sclerosis and cortical erosion. In the erosive area of the bone, a corresponding edema (**b**,**c**) can be observed as well as increased gadolinium enhancement (**d**). [18F]fluoride-PET/CT (**e**) and [18F]FDG-PET/MRI (**f**) reveal markedly increased tracer uptake in the OM affected jawbone below the extent of sclerosis.

**Figure 3:**
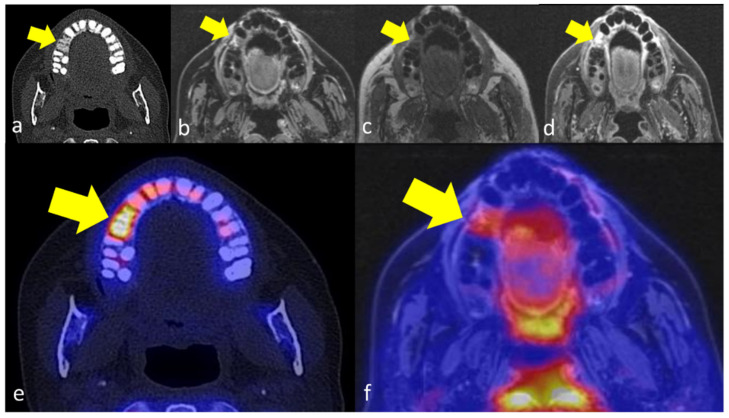
49 years old female patient with secondary chronic OMJ in region 15 of the maxilla (yellow arrows). The OM affected jawbone shows a diffuse trabecular sclerosis in CT (**a**), an edema in T2-weighted images (**b**) and corresponding T1-weighted images (**c**) with increased contrast enhancement after gadolinium application (**d**). The corresponding PET images show a markedly increased [18F]fluoride uptake in the affected jawbone (**e**) and an increased [18F]FDG-uptake (**f**).

The affected jawbone was characterized by trabecular sclerosis on CT as compared to healthy jawbone in all 6 patients, while additional cortical erosions were observed in 3/6 patients (Figure 1 and Figure 2). In MRI, bone marrow edema and increased gadolinium enhancement were both documented in 5/6 patients. The extent of affected regions in MRI was smaller compared to CT in three patients and equal to CT in two patients. The periosteal soft tissue adjacent to the affected jawbone was characterized by increased gadolinium enhancement in all cases. On PET imaging, increased [18F]fluoride uptake was observed in the affected jawbone compared to the healthy jawbone in all five patients (Figure 1, Figure 2 and Figure 3).

The extent of affected regions in the jawbone where increased metabolic activity was observed on [18F]fluoride-PET was smaller than the morphologic changes on CT in all cases and smaller than the morphologic changes on MRI in 3/4 patients who could be compared in this setting. In one patient, the extent of affected regions with increased [18F]fluoride-uptake was greater than on MRI (Figure 2).

In [18F]FDG-PET, the extent of affected regions with increased PET tracer uptake was lower compared to CT and MRI in 4/5 patients, while one patient showed an equal distribution pattern of increased PET tracer uptake and morphologic changes in jawbone. In 3/5 of the patients who underwent [18F]FDG-PET/MRI, [18F]FDG uptake was only moderately increased in the affected jawbone and adjacent tissue (Figure 1e, Figure 2e and Figure 3e). Table 1 summarizes the extent of OMJ in each imaging modality.

### 3.3. Quantitative Analysis

The mean HU in the trabeculae of the affected jawbone was higher compared to the healthy jawbone (560 ± 328 HU vs. 282 ± 211 HU, *p* > 0.05). According to bone edema, T2 signal was higher in affected jawbone (113 ± 101) compared to healthy jawbone (37 ± 16, *p* > 0.05) and T1 signal decreased (211 ± 121 vs. 296 ± 127, *p* > 0.05). After gadolinium injection, post-contrast T1 signal was significantly higher in the affected jawbone (464 ± 174) compared to the healthy jawbone (237 ± 98, *p* < 0.05).

[18F]fluoride uptake was significantly higher in the affected jawbone than in the healthy jawbone (15.4 ± 4.2 vs. 2.1 ± 0.6, *p* < 0.01). [18F]FDG uptake was moderately but significantly higher in the affected jawbone than in the healthy jawbone (1.9 ± 0.7 vs. 0.7 ± 0.2, *p* < 0.01) which was also observed in the adjacent peri-mandibular soft tissue (2.4 ± 0.8 vs. 1.9 ± 0.4, *p* > 0.05). Detailed results are shown in Table 2.

## 4. Discussion

There is still no gold standard for diagnosis of OMJ [1,24,25]. We investigated a diagnostic workup using [18F]FDG-PET/MRI and [18F]fluoride-PET/CT in a cohort of patients with symptomatic OMJ to evaluate both, morphologic and metabolic changes of the jawbone. This proof-of-principle study provides first results in terms of delineating the extent of active disease, which cannot be determined by conventional CT or MRI alone as changes in trabecular structure and cortical integrity do not always correspond to functional information about bone metabolism and turnover.

The diagnosis was made in all patients in our cohort primarily by recurrent pain as well as local swelling and reduced mouth opening, which are regularly observed clinical symptoms in patients with OMJ [1]. Diagnosis was also confirmed by the fact that treatment strategies were not effective for OMJ other than primary chronic OMJ. In 5/6 patients of our cohort, the mandible was the affected bone of OMJ. This is consistent with the literature, which describes that OMJ is most commonly diagnosed in the mandible and usually affects the mandibular body, followed by the symphysis, angle, ascending ramus, and condyle [4,28].

In our study, OM affected jawbone was associated with trabecular edema as a correlate of inflamed tissue leading to exudate, hyperemia, and bone ischemia [29]. Similarly, we observed increased gadolinium enhancement in the affected jawbone and increased trabecular sclerosis compared with healthy jawbone. Interestingly, we documented a markedly increased [18F]fluoride uptake in parts of the affected jawbone, which extended to fewer regions than the morphologic findings on CT and MRI.

The fact that chronic inflammation in the skeleton is associated with the replacement of normal bone marrow by fibrotic tissue may explain our observation that the findings on CT and MRI exceeded the areas of increased [18F]fluoride uptake in all patients [5]. [18F]fluoride is incorporated into hydroxyapatite by osteoblasts during new bone formation and therefore allows detection of subtle foci of increased bone remodeling [30]. After intravenous injection, [18F]fluoride is rapidly excreted from the plasma and incorporated into the bone crystal structure of bone by passive diffusion. [18F]fluoride has the advantage of not being affected by bone marrow activity, as this radiotracer mainly represents cortical osteoblastic function [31]. In addition, [18F]fluoride is not affected by glycemic status of patients, suggesting that the standardized uptake value (SUV) of [18F]fluoride varies less than the SUV of [18F]FDG [32]. Several investigators have used [18F]fluoride PET to quantitatively assess bone turnover since [18F]fluoride uptake correlates with bone histomorphometry [33]. Studies suggest that [18F]fluoride PET is useful in several benign bone diseases such as osteonecrosis [15], avascular necrosis [34], condylar hyperplasia [35], prosthetic loosening, or atypical foot pain [36,37].

The development of acute to secondary chronic osteomyelitis can be divided into the following phases: microbial invasion and biofilm proliferation, immune response, and the impact of bacterial invasion on bone tissue components [38]. The inflammatory response may culminate in pus production, increasing the intramedullary pressure and reducing blood flow into the jawbone. Granulation tissue is characterized by edema and usually shows increased gadolinium enhancement, although this finding may be negligible in secondary OMJ [39,40]. Bone sclerosis and medullary fibrosis are common in OMJ [41].

Our results suggest that the jawbone regions with increased [18F]fluoride uptake are more likely to represent the extent of active disease than CT or MRI.

We observed moderately increased [18F]FDG uptake in the affected jawbone and adjacent perimandibular soft tissue, which was consistent with the extent of affected regions on [18F]fluoride PET. [18F]FDG is transported across the cell membrane by glucose transport proteins and accumulates at sites of infection and inflammation, which is why it is used in chronic osteomyelitis. Neutrophils, plasma cells, histiocytes, and lymphocytes may be present in OMJ to varying degrees, resulting in different amounts of inflammatory cells in the bone marrow. Active osteoclasts are found in 96% of samples [42]. The extent of [18F]FDG uptake is therefore an indicator of subacute inflammatory changes in the jawbone during the course of OMJ. For correct depiction of the presence or absence of chronic osteomyelitis, [18F]FDG-PET has a sensitivity of 100% and a specificity of 87.5% [43]. However, positive PET findings may be observed due to other causes leading to inflammation of the bone or surrounding soft tissue [43].

Our results support previous findings that [18F]fluoride does not accumulate in acute inflammatory processes and only indicates the area of active bone remodeling. Since the morphologic changes of the jawbone depicted by CT or MRI remarkably exceeded the functional changes in [18F]fluoride, the combination of both imaging modalities may allow a more objective classification of the actual disease activity. This information, in combination with the amount of [18F]FDG uptake, could be a valuable decision-making tool for therapy approaches in symptomatic patients. Of course, our results need to be confirmed in larger patient collectives with long-term clinical observation. In our study, all patients received systemic antibiotic treatment after imaging, which resulted in symptom relief in 4/6 patients.

As mentioned earlier, a major limitation of the study is the small size of the cohort. This justifies the implementation of larger prospective studies that include long-term outcome analyses. However, the primary chronic form of OMJ in particular is a rare disease. To our knowledge, this is the first study comparing imaging features of OMJ with [18F]fluoride PET, [18F]FDG-PET, CT, and MRI. Despite of the lack of a gold standard for reliable diagnosis, most patients in our cohort could be assigned relatively reliably. Imaging as part of the study protocol was performed only for therapy planning when patients had diffuse symptoms to support differentiation between active and inactive OMJ. However, we did not acquire follow-up imaging data as part of the study that would allow direct comparison after treatment.

## 5. Conclusions

In conclusion, information obtained from [18F]FDG-PET and [18F]fluoride-PET on jawbone metabolism and inflammation is different from morphologic changes and therefore has the potential to provide a more accurate and objective assessment of the extent and activity of OMJ. This could support therapeutic decisions in a clinical setting.

## Figures and Tables

**Table 1:** Extent of OMJ depending on imaging modality.

	CT		MRI		[^18^F]FDG PET		[^18^F]Fluorid PET	
	Trabecular Sclerosis (Regions)	Cortical Erosion (Regions)	Bone Marrow Edema (Regions)	Gadolinium Enhancement (Regions)	Regions	SUV_mean_	Regions	SUV_mean_
**Patient 1**	34–42	32–33	31–33	31–33	31	3.7	/	/
**Patient 2**	LMR–42	n.o.	n.o.	n.o.	/	/	41, 36–37	10.6
**Patient 3**	43–47	n.o.	44–47	43–48	46	0.7	45–47	18.8
**Patient 4**	14–15, 46–47	n.o.	14–15, 46–47	14–15, 46–47	14–15	0.9	14–15, 47	13.0
**Patient 5**	43–RMC	RMC	RMC	RMC	RMC	2.3	RMR–RMC	37.7
**Patient 6**	37–48	32–33	33–43	33–43	33	2.0	33–42	14.5

Abbreviations: n.o., not observed; LMR, left mandibular ramus; RMR, right mandibular ramus; RMC, right mandibular condyle.

**Table 2:** Quantitative imaging markers in affected jawbone and healthy jawbone. Indicated as Mean ± SD.

Imaging Markers	Affected Bone	Healthy Bone	SQI	*p* Value
**T2w**	113 ± 101	37 ± 16	3.2 ± 2.1	>0.05
**T1w**	211 ± 127	296 ± 127	0.9 ± 0.5	>0.05
**T1w post-contrast (bone)**	464 ± 174	237 ± 98	2.4 ± 1.3	<0.05
**^18^F-FDG PET SUV_mean_ (bone)**	1.9 ± 0.7	0.7 ± 0.2	2.6 ± 0.6	<0.05
**^18^F-FDG PET SUV_mean_ (soft tissue)**	2.4 ± 0.8	1.9 ± 0.4	1.3 ± 0.3	>0.05
**^18^F-fluoride PET SUV_mean_ (bone)**	15.4 ± 4.2	2.1 ± 0.6	7.4 ± 1.3	<0.01
**Hounsfield Units**	560 ± 328	282 ± 211	2.2 ± 0.9	>0.05

SQI, semi-quantitative index.

## Data Availability

The data presented in this study are available on request from the corresponding author.
